# Describing the burden of moderate exacerbations in patients with asthma from the Extended Salford Lung Study (Ext-SLS): a retrospective cohort study

**DOI:** 10.1186/s12931-025-03199-5

**Published:** 2025-03-29

**Authors:** Emma Goodall, Kieran J. Rothnie, Beade Numbere, Shiyuan Zhang, Chris Compton, Robert Wood, Theo Tritton, Rosie Wild, Mark Small, Jørgen Vestbo, Ashley Woodcock

**Affiliations:** 1 Epidemiology, Organisation of the Chief Medical Officer, GSK R&D, London, UK; 2 RWE&HOR, Organisation of the Chief Patient Officer, GSK, Collegeville, PA USA; 3 Global Medical Affairs,General Medicines, GSK, London, UK; 4 Real-world Evidence, AdelphiRealWorld, Bollington, Cheshire, UK; 5 University of Manchester, Manchester, UK; 6 Wythenshawe Hospital, Manchester, UK; 7 Global Epidemiology, Organisation of the Chief Medical Officer, GSK R&D, London, UK

**Keywords:** Asthma, Dual therapy, Electronic health record, Healthcare resource utilisation, Hospital episode statistics, Inhaled corticosteroids, Long-acting β_2_-agonists, Moderate exacerbations, Patient-reported outcomes, Salford lung study

## Abstract

**Background:**

There is a need for real-world data describing the frequency and impact of moderate asthma exacerbations in patients receiving inhaled corticosteroids/long-acting β_2_-agonists (ICS/LABA). The Salford Lung Study (SLS) and associated extension study (Ext-SLS) evaluated ICS/LABA versus existing maintenance therapy in adults with asthma. This analysis assessed the impact of moderate exacerbations in patients from the Ext-SLS.

**Methods:**

This retrospective cohort study analysed linked primary and secondary care and patient questionnaire data from patients enrolled in the Ext-SLS (indexed April 2018–May 2019). Primary outcome was number of self-reported moderate asthma exacerbations 12 months pre-index, overall, by maintenance treatment class and asthma control status at index, using the Asthma Control Test (ACT; poor [< 16], somewhat controlled [16–18], and controlled [> 19]) and 6-item Asthma Control Questionnaire (ACQ-6; uncontrolled [≥ 1.50], partially controlled [> 0.75–<1.50], and controlled [≤ 0.75]). Secondary outcomes included index ACT and ACQ-6 score, healthcare resource utilisation (HCRU) and direct costs 12 months pre- and post-index, stratified by self-reported moderate exacerbation frequency pre-index.

**Results:**

Of 485 patients with ≥ 12 months’ pre-index data, 86.6% (n = 420) self-reported moderate exacerbations, with similar frequency irrespective of maintenance treatment class (66.7–100.0%; ICS/LABA: 85.4%). Numerically greater proportions of patients self-reported a moderate exacerbation in the 12 months pre-index in ACT poor-control (n = 110/115 [95.7%]) and ACQ-6-uncontrolled (n = 200/210 [95.3%]) versus ACT- and ACQ-6-controlled (n = 205/260 [78.8%], n = 105/145 [72.4%]) groups. Symptom control worsened with increasing exacerbation frequency: mean (SD) ACT scores were 21.8 (3.3) and 15.7 (4.4) for patients with 0 or ≥ 7 events, respectively; mean (SD) ACQ-6 scores followed the same trend. Direct costs and HCRU increased with pre-index exacerbation frequency; mean (SD) all-cause and asthma-related total costs were £1509 (£2384) and £717 (£1459) for patients with no moderate exacerbations 12 months pre-index and £2002 (£2058) and £1086 (£1538) for patients with ≥ 7 exacerbations; similar trends occurred over 12 months post-index.

**Conclusions:**

Patients with asthma experience frequent moderate exacerbations, which are associated with poor asthma control, increased HCRU and costs, emphasising the poor quality of life patients experience. Tackling poor adherence, risk behaviour, and comorbidities as well as holistic management and medication review are needed.

**Clinical trial details:**

Registered on clinicaltrials.gov: NCT03152669, 12 May 2017

**Supplementary Information:**

The online version contains supplementary material available at 10.1186/s12931-025-03199-5.

## Background

Asthma exacerbations, characterised by episodes of chest tightness, shortness of breath, and a decline in lung function, can occur in a range of severities [1]. Moderate exacerbations may require a change in treatment to prevent the exacerbation from worsening, while severe exacerbations typically necessitate urgent initiation of systemic corticosteroids and can result in hospitalisation or death [2]. Several studies have established that severe asthma exacerbations incur a substantial burden, such as negative impacts on patient quality of life, frequent hospitalisations, and high healthcare-related costs [3–5]. A limited number of previous studies looking at moderate exacerbations alone have shown them to be associated with a negative effect on patients’ lives [6], and they can lead to life-threatening severe events [7]. Additionally, the fact that moderate exacerbations occur more frequently than severe exacerbations [8] suggests that their adverse impacts are likely to be a more common experience for patients.

Since 2019, the Global Initiative for Asthma report recommends combined low-dose inhaled corticosteroid (ICS)/long-acting β_2_-agonist (LABA; formoterol) therapy as needed at treatment Steps 1–2; and as maintenance and reliever therapy at Step 3 and above [1, 9] and pre-2019, recommended low-dose ICS/LABA therapy maintenance therapy with as-needed short-acting β_2_-agonist (SABA) or ICS/formoterol at Step 3 [9]. Despite these recommendations, between 28% and 80% of patients are estimated to have inadequately controlled asthma despite ICS/LABA therapy [10–14], leading to high levels of symptom burden, increased rescue medication use, health-related quality of life impairment, and an increased frequency of exacerbations [11, 15, 16]. Given the currently limited evidence for the impact of moderate exacerbations, there is a need for further real-world data describing the frequency and burden of moderate asthma exacerbations in patients with asthma, as well as among those whose asthma remains uncontrolled despite ICS/LABA therapy.

The Salford Lung Study (SLS) was a large, pragmatic, randomised controlled trial that evaluated the clinical effectiveness and safety of once-daily fluticasone furoate (100–200 µg)/vilanterol (25 µg) in a dry-powder inhaler versus existing maintenance therapy in adult patients with asthma living in a predominantly deprived urban area in the United Kingdom (UK) [17]. The SLS extension study (Ext-SLS) expanded access to patients’ healthcare data and included retrospective primary and secondary care electronic medical record (EMR) data, as well as questionnaire data relating to asthma history, triggers and management, tobacco exposure, self-reported exacerbations and asthma control, aspects not routinely available in EMR data, allowing for the observation of patients’ entire disease course [18]. Using these real-world data, the objective of this study was to quantify the frequency and burden of moderate exacerbations experienced by patients with asthma who participated in the Ext-SLS, as well as in the subgroup of patients using ICS/LABA.

## Methods

### Study design

This was a retrospective cohort study (GSK study 214485, registered on clinicaltrials.gov: NCT03152669, 12 May 2017), analysing linked UK primary care EMR data and secondary care Hospital Episode Statistics (HES) data in England, and Ext-SLS patient questionnaire data from patients with asthma. The indexing period was between April 2018 and May 2019, with the index date defined as the date on which each patient consented to inclusion in the Ext-SLS (Additional file 1: Figure S1). To ensure patients had both primary and secondary care data, patient observation began at the start of either primary care or secondary care data availability in April 2009, whichever was later. Patients were followed until study end or death.

Primary care EMR data included non-sensitive primary demographic healthcare records collected directly from general practitioners (GPs) until December 2019. Primary care data were obtained direct from source and contributed by GP practices using both EMIS^®^ and Vision^®^ software. Secondary care HES data in England spanned from April 2009 at the earliest until March 2020. The Ext-SLS questionnaire was self-completed by patients at index, and was designed to capture asthma history, triggers and management, self-reported exacerbations, tobacco exposure, and patient-reported outcome measures (PROMs), including the Asthma Control Test (ACT), the 6-item Asthma Control Questionnaire (ACQ-6), and the Chronic obstructive pulmonary disease (COPD) and Asthma Sleep Impact Scale (CASIS).

### Study population

Patients eligible for inclusion in the Ext-SLS needed to have re-consented to additional data collection, to have been randomised to treatment in the original SLS and have four primary care visits recorded and matched with SLS trial data for identification purposes. Excluded patients included those who were withdrawn from the SLS, were unable to provide consent for the Ext-SLS, were lost to follow-up, had died, or were deemed too ill to participate by their GP, as well as those unable to be identified as an original SLS patient (e.g., those no longer registered at the same GP practice as during the SLS).

Patients from the Ext-SLS who were eligible for inclusion in this study needed to have primary care EMR data available, have provided valid responses to two asthma management sections of the Ext-SLS questionnaire (number of extra inhalations taken during a moderate asthma exacerbation and number of moderate asthma exacerbations in the previous year), and have both ACT and ACQ-6 scores recorded.

All patients were eligible for linkage to secondary care (HES) EMR data pre-index (through to study end), so no restrictions based on availability of EMR data during the study period were required, nor were any other exclusion criteria applied for this specific study.

### Outcomes

The primary outcome was the number of self-reported moderate asthma exacerbations in the 12 months pre-index. Moderate asthma exacerbations were defined as events requiring an increase in rescue/reliever therapy, but which did not require treatment with systemic steroids or accident and emergency (A&E)/hospital attendance. Severe exacerbations were defined as events requiring A&E/hospital attendance or use of systemic steroids. Moderate asthma exacerbation data were described for the overall population, by maintenance treatment class at index (± 15 days) and asthma control status at index, which was assessed using the ACT and ACQ-6 (recall period of 4 weeks and 1 week, respectively). Treatment class subgroups at index included patients receiving short-acting bronchodilators only, ICS only, ICS/LABA, multiple-inhaler triple therapy (MITT), ICS + leukotriene receptor antagonist (LTRA) + long-acting muscarinic antagonist (LAMA) or LABA, ICS/LABA + LAMA + LTRA, other maintenance therapies or none. Asthma control subgroups based on ACT score (range 5–25) were poor control (< 16), somewhat controlled (16–19), and controlled (> 19); ACQ-6 (range 0–6) subgroups were defined as uncontrolled (≥ 1.50), partially controlled (> 0.75–<1.50), and controlled (≤ 0.75).

Secondary outcomes included patient clinical, and asthma-related characteristics in the 12 months pre-index (including ACT and ACQ-6 score at index), all-cause and asthma-related healthcare resource utilisation (HCRU) and direct healthcare costs in the 12 months pre- and post-index. Outcomes were reported for the overall population and stratified by the number of self-reported moderate exacerbations (0, 1–3, 4–6 and ≥ 7) in the 12 months pre-index, and for the subset of patients using ICS/LABA maintenance therapy. ICS/LABA use was defined as ≥ 1 prescription of ICS/LABA with ≥ 1 day of coverage in the 3 months pre-index.

Total HCRU and direct costs were assessed and included GP, outpatient, inpatient (including length of stay), and A&E visits. Direct healthcare costs also included costs for asthma medications prescribed in primary care prescriptions. Costs (£) were calculated via the application of national tariffs to derived healthcare resource groups (HRGs) for each secondary care interaction/service use. The cost of primary care GP or nurse consultations reflected the Unit Costs of Health and Social Care 2019 document from the University of Kent’s Personal Social Services Research Unit [19]. Medication costs were based on the 2019 National Health Service (NHS) Drug Tariff, compiled and provided by NHS Prescription Services [20], or from the British National Formulary [21] when unit costs could not be sourced from the Drug Tariff. Reimbursement costs for particular HRG codes including outpatient visits, hospitalisations, critical care admissions and A&E visits were derived from the HRG4 + 2019/20 Local Payment Grouper [22].

### Statistical analyses

As this was a descriptive study, no formal statistical analysis was conducted. Continuous variables were described using means (standard deviation [SD]) and medians (interquartile range [IQR]); frequencies and proportions were used to describe categorical variables. Two overall populations were defined: (1) patients with ≥ 12 months pre-index data and (2) patients with both ≥ 12 months pre- and post-index data, to assess HCRU and healthcare costs over time.

To account for any potential data limitations, three sensitivity analyses were performed on the primary outcome. The first excluded patients with comorbid COPD (determined by the presence of diagnosis codes pre-index [inclusive]). The second and third sensitivity analyses addressed data from those with an invalid response (“I don’t use more reliever inhaler” or “Don’t know/no answer”) in the Ext-SLS questionnaire for the number of extra inhalations taken during a moderate asthma exacerbation. For how often this occurred during the previous 12 months, the second analysis imputed a value of ‘0’ whereas the third analysis imputed values of 0 or 1–3 for patients with ACT scores that indicated “controlled” symptoms (ACT > 19) and “not controlled” symptoms (ACT ≤ 19), respectively.

When reporting outcomes, results based on fewer than eight patients were suppressed to protect patient confidentiality, and all other results were rounded to the nearest five to protect primary suppression in accordance with HES analysis guidance [23]. All analyses were conducted using Stata 17 software, version 17 (StataCorp, College Station, TX).

## Results

Of 815 patients from the Ext-SLS with available data, 485 met the eligibility criteria and had ≥ 12 months of pre-index data; of those, 450 patients also had ≥ 12 months of post-index data (Additional file 2: Figure S2).

### Self-reported moderate exacerbations

**Fig. 1:**
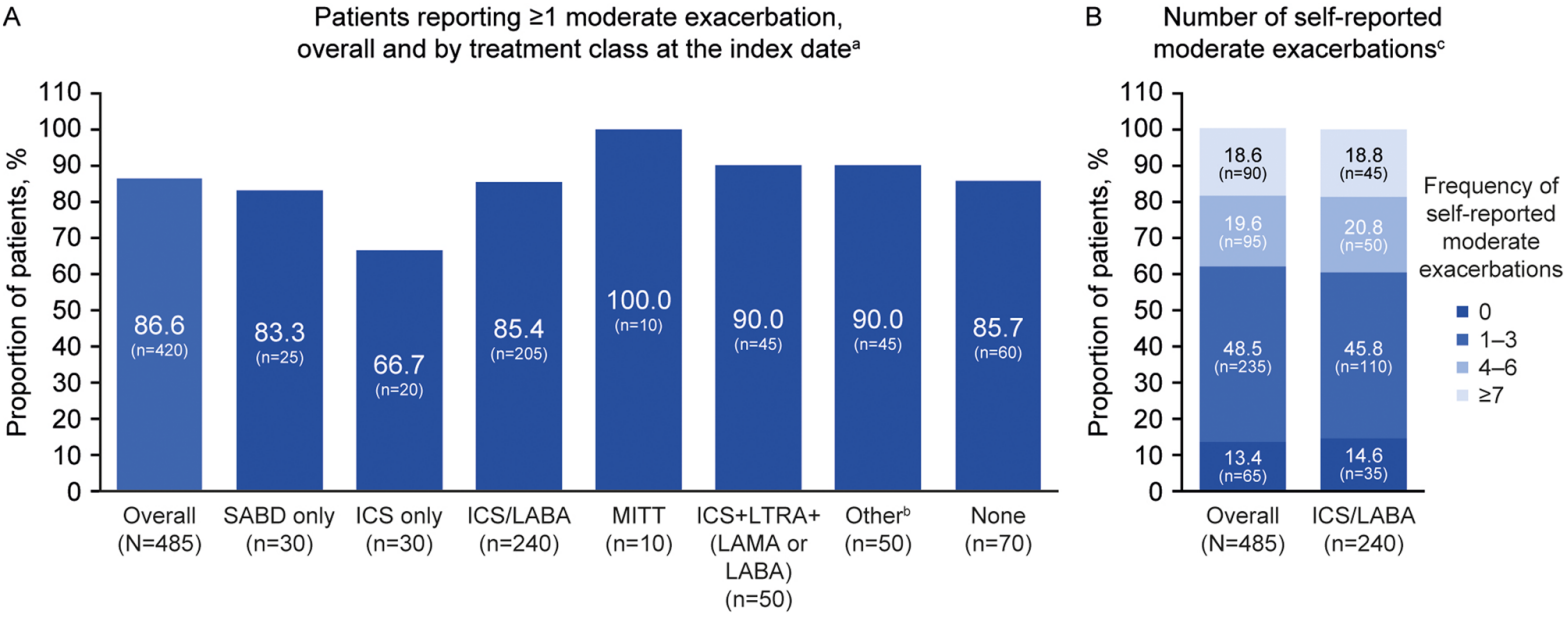
Self-reported moderate exacerbations (pre-index) overall, by treatment class (**A**) and in ICS/LABA users (**B**). Self-reported moderate exacerbations were assessed during the 12 months pre-index (date of consent to inclusion in the Ext-SLS). Results based on 1–<8 patients were suppressed, and all other counts were rounded to the nearest five to comply with HES analysis guidance [23]; consequently, proportions of patients may not total 100%. ^a^Data were unavailable for ICS + LTRA, SITT, and ICS/LABA + LAMA + LTRA due to data suppression; ^b^including OCS monotherapy, methylxanthines and PDE4 inhibitors, as well as non-standard asthma maintenance regimens including ICS, LABA or LTRA monotherapy, and combinations of ICS, LABA, LAMA, LTRA, OCS, SABD and xanthines. Free/open combination therapy was identified when there was ≥ 1 day of overlap between all components; ^c^ICS/LABA use was defined as a prescription of fixed-dose combination ICS/LABA at index (± 15 days). Ext-SLS, Extended Salford Lung Study; HES, Hospital Episode Statistics; ICS, inhaled corticosteroid; LABA, long-acting β_2_-agonist; LAMA, long-acting muscarinic antagonist; LTRA, leukotriene receptor antagonist; MITT, multiple-inhaler triple therapy; OCS, oral corticosteroid; PDE4, phosphodiesterase-4; SABD, short-acting bronchodilator; SITT, single-inhaler triple therapy

Of the 485 patients, 86.6% (*n* = 420) had a self-reported moderate exacerbation in the 12 months pre-index (Fig. 1A); 48.5% (*n* = 235) had 1–3 exacerbations, 19.6% (*n* = 95) had 4–6 exacerbations, and 18.6% (*n* = 90) had ≥ 7 exacerbations (Fig. 1B).

### Stratified by asthma maintenance class

For patients receiving ICS/LABA at index (*n* = 240), 85.4% (*n* = 205) self-reported ≥ 1 moderate exacerbation, 45.8% (*n* = 110) had 1–3 exacerbations, 20.8% (*n* = 50) had 4–6 exacerbations and 18.8% (*n* = 45) had ≥ 7 moderate exacerbations in the 12 months pre-index (Fig. 1B). The proportion of patients self-reporting ≥ 1 moderate exacerbation in the 12 months pre-index for other asthma maintenance classes was 66.7% (*n* = 20/30) for patients receiving ICS-only maintenance therapy, 100% (*n* = 10/10) for those taking MITT, and 90% (*n* = 45/50) for those taking either ICS + LTRA + LAMA or LABA or “other” therapies (Fig. 1A).

### Stratified by asthma control status

**Fig. 2:**
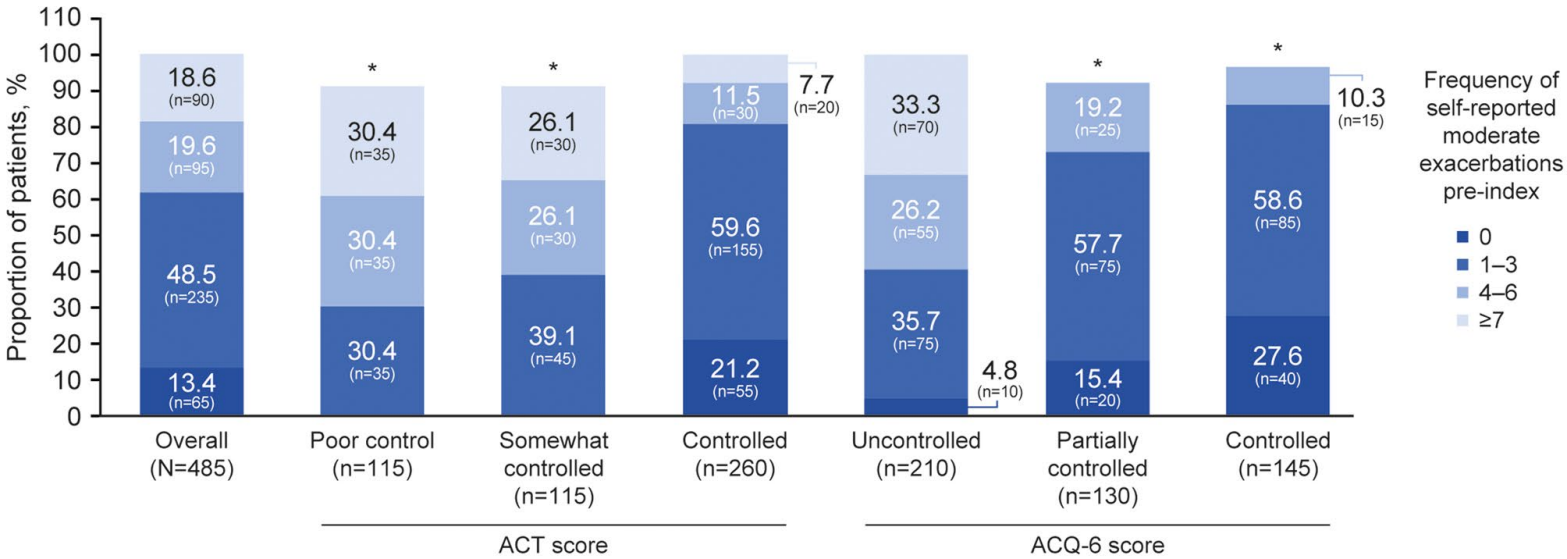
Self-reported moderate exacerbations (pre-index), by asthma control status. (ACT and ACQ-6) at index. Self-reported moderate exacerbations were assessed 12 months pre-index (date of consent to inclusion in the Ext-SLS). Results based on 1–<8 patients were suppressed (denoted by an asterisk), and all other counts were rounded to the nearest five to comply with HES analysis guidance [23]; consequently, proportions of patients may not total 100%. ACT score: poor control (< 16), somewhat controlled (16–19), and controlled (> 19). ACQ-6 score: uncontrolled (≥ 1.50), partially controlled (> 0.75 to < 1.5), and controlled (≤ 0.75). ACQ-6, Asthma Control Questionnaire 6-item; ACT, Asthma Control Test; Ext-SLS, Extended Salford Lung Study; HES, Hospital Episode Statistics

The frequency of self-reported moderate exacerbations pre-index increased with worsening asthma control status (Fig. 2). Among patients classified as controlled according to ACT scores, 59.6% (*n* = 155/260) self-reported 1–3 moderate exacerbations, compared with 30.4 (*n* = 35/115) classified as poorly controlled. Conversely, the proportion of patients self-reporting 4–6 moderate exacerbations (30.4% [*n* = 35/115] vs. 11.5% [*n* = 30/260]) and ≥ 7 moderate exacerbations (30.4% [*n* = 35/115] vs. 7.7% [*n* = 20/260]) was higher among poorly controlled versus controlled patients (Fig. 2). Similar results were seen when asthma control status was defined using ACQ-6 scores (Fig. 2).

Similar trends were seen across all stratifications of the primary outcome when sensitivity analyses were performed (Additional file 3: Figure S3 and Additional file 4: Figure S4).

### Sociodemographic, clinical and asthma-related characteristics

Overall, mean (SD) age at index date was 55.5 (14.3) years, with the mean age being generally similar across all self-reported moderate exacerbation subgroups (Table 1). The 28.9% (*n* = 140/485) of patients classified in the ‘most deprived’ quintile had the highest frequency of self-reported exacerbations in the 12 months pre-index across all exacerbation groups compared with the other deprivation status groups, with the proportion of patients in the ‘most deprived’ quintile increasing with higher exacerbation frequency (Table 1). Patients with ≥ 7 moderate exacerbations pre-index had a numerically higher body mass index than those with no moderate exacerbations. There was a trend for a decrease in the proportion of non-smokers as pre-index exacerbation frequency increased. Lung function was generally similar for all exacerbation frequencies, although patients with ≥ 7 exacerbations had the lowest peak expiratory flow rate (PEFR) (Table 1).

**Table 1:** Sociodemographic, clinical, and asthma-related characteristics

Characteristic	Overall (*N* = 485)	Frequency of self-reported moderate asthma exacerbations in the 12 months pre-index				ICS/LABA users in the 3 months pre-index (*n* = 365)
		0 (*n* = 65)	1–3 (*n* = 235)	4–6 (*n* = 95)	≥ 7 (*n* = 90)	
**Age at index date**,** years**,** mean (SD)**	55.5 (14.3)	53.0 (15.8)	54.8 (14.1)	57.0 (14.1)	57.8 (13.4)	56.7 (14.2)
**Female**,** n (%)**	325 (67.0)	45 (69.2)	160 (68.1)	65 (68.4)	55 (61.1)	250 (68.5)
**Deprivation level of local area**^**a**^**at index**,** n (%)**						
Most deprived	140 (28.9)	< 8	65 (27.7)	35 (36.8)	35 (38.9)	110 (30.1)
More deprived	65 (13.4)	< 8	30 (12.8)	10 (10.5)	15 (16.7)	50 (13.7)
More/Less deprived	80 (16.5)	15 (23.1)	40 (17.0)	20 (21.1)	10 (11.1)	55 (15.1)
Less deprived	75 (15.5)	15 (23.1)	35 (14.9)	10 (10.5)	15 (16.7)	60 (16.4)
Least deprived	60 (12.4)	15 (23.1)	30 (12.8)	10 (10.5)	10 (11.1)	45 (12.3)
Unknown	70 (14.4)	10 (15.4)	35 (14.9)	15 (15.8)	10 (11.1)	50 (13.7)
**BMI (kg/m** ^**2**^ **) two years pre-index (inclusive)**						
n	430	60	210	85	75	330
Mean (SD)	30.7 (7.3)	28.1 (28.1)	31.2 (31.2)	29.9 (7.7)	32.1 (7.4)	30.6 (7.3)
**Self-reported smoking status at index**,** n (%)**						
Current smoker	60 (12.4)	< 8	25 (10.6)	10 (10.5)	20 (22.2)	50 (13.7)
Former smoker	185 (38.1)	20 (30.8)	90 (38.3)	40 (42.1)	35 (38.9)	140 (38.4)
Non-smoker	230 (47.4)	40 (61.5)	120 (51.1)	40 (42.1)	30 (33.3)	170 (46.6)
Unknown	< 8	0 (0.0)	< 8	< 8	< 8	< 8
**Comorbidities 12 months ** **pre-index (inclusive)**,** n (%)**						
Pneumonia (ever)	235 (48.5)	30 (46.2)	110 (46.8)	45 (47.4)	50 (55.6)	175 (47.9)
Atopy	185 (38.1)	25 (38.5)	95 (40.4)	30 (31.6)	35 (38.9)	140 (38.4)
Depression	185 (38.1)	15 (23.1)	90 (38.3)	35 (36.8)	45 (50.0)	145 (39.7)
Anxiety	115 (23.7)	10 (15.4)	55 (23.4)	25 (26.3)	25 (27.8)	90 (24.7)
GORD	110 (22.7)	10 (15.4)	45 (19.1)	25 (26.3)	30 (33.3)	90 (24.7)
COPD^b^	80 (16.5)	10 (15.4)	30 (12.8)	25 (26.3)	20 (22.2)	65 (17.8)
Diabetes mellitus	70 (14.4)	< 8	25 (10.6)	20 (21.1)	20 (22.2)	55 (15.1)
Coronary artery disease	70 (14.4)	< 8	30 (12.8)	15 (15.8)	20 (22.2)	55 (15.1)
Pneumonia (in previous year)	50 (10.3)	< 8	25 (10.6)	10 (10.5)	10 (11.1)	35 (9.6)
Arrhythmia	50 (10.3)	< 8	15 (6.4)	10 (10.5)	20 (22.2)	40 (11.0)
Chronic rhinosinusitis	45 (9.3)	< 8	25 (10.6)	10 (10.5)	< 8	35 (9.6)
**PEFR %**,** two years pre-index (inclusive)**						
n	360	50	175	70	60	280
Mean (SD)	90.5 (17.0)	91.0 (13.5)	93.0 (16.1)	90.1 (16.7)	83.2 (20.6)	90.8 (17.1)
**Asthma symptoms 12 months pre-index (inclusive)**,** n (%)**						
None	360 (74.2)	65 (100.0)	175 (74.5)	60 (63.2)	60 (66.7)	270 (74.0)
Any	125 (25.8)	< 8	60 (25.5)	35 (36.8)	25 (27.8)	95 (26.0)
Cough (non-acute)	70 (14.4)	< 8	40 (17.0)	15 (15.8)	15 (16.7)	55 (15.1)
Night-time awakening	35 (7.2)	< 8	10 (4.3)	15 (15.8)	< 8	25 (6.8)
Breathlessness	25 (5.2)	0 (0.0)	10 (4.3)	< 8	< 8	20 (5.5)
Chest tightness	15 (3.1)	< 8	10 (4.3)	0 (0.0)	< 8	10 (2.7)
Wheeze	10 (2.1)	< 8	< 8	< 8	< 8	10 (2.7)
Fatigue	< 8	0 (0.0)	0 (0.0)	0 (0.0)	< 8	< 8
**Eosinophil count 2 years ** **pre-index (inclusive) (cells/µL)**						
n	305	30	145	60	70	235
Geometric mean (log SD)	0.167 (0.9)	0.144 (1.1)	0.169 (0.9)	0.174 (0.8)	0.170 (0.9)	0.166 (0.9)
**SABA prescriptions**						
Patients with SABA prescription, n/N (%)	395/450 (90.8)	55/65 (91.7)	185/220 (88.1)	80/85 (94.1)	80/80 (100)	305/340 (89.7)
Mean (SD) prescriptions	5.0 (4.2)	3.2 (3.2)	4.6 (4.1)	5.1 (4.1)	7.3 (4.4)	5.4 (4.4)
Median (IQR) prescriptions	4.0 (2.0, 7.0)	2.0 (1.0, 5.0)	3.0 (1.0, 7.0)	4.0 (2.0, 7.0)	6.0 (4.0, 11.0)	4.0 (2.0, 8.0)

Results based on 1–<8 patients were suppressed, and all other counts were rounded to the nearest five to comply with HES analysis guidance [23]; consequently, proportions of patients may not total 100%. Index was defined as the date on which each patient consented to inclusion in the Ext-SLS. ^a^Deprivation level of local area was described according to English Index of Multiple Deprivation quintiles [39], or Unknown; ^b^including emphysema and chronic bronchitis

BMI, body mass index; COPD, chronic obstructive pulmonary disease; Ext-SLS, Extended Salford Lung Study; GORD, gastro-oesophageal reflux disease; HES, Hospital Episode Statistics; ICS, inhaled corticosteroid; IQR, interquartile range; LABA, long-acting β_2_-agonist; PEFR, peak expiratory flow rate; SABA, short-acting β_2_-agonist; SD, standard deviation

Overall, the most frequent comorbidity observed across all self-reported moderate exacerbation subgroups was pneumonia (48.5%, *n* = 235/485), followed by depression and atopy (38.1%, *n* = 185/485 for both) (Table 1). In general, a greater proportion of patients who self-reported ≥ 7 exacerbations in the 12 months pre-index reported comorbidities compared with the other exacerbation groups. Overall, 74.2% (*n* = 360/485) of patients had no asthma symptoms in the 12 months pre-index (inclusive); of those with symptoms, the most common were cough (non-acute) (14.4%, *n* = 70/485) and night-time awakening (7.2%, *n* = 35/485) (Table 1). The proportion of patients with any asthma symptom was similar across the exacerbation frequency categories (25.5–27.8%), except for 4–6 moderate exacerbations (36.8%) (Table 1).

Patients who were ICS/LABA users in the 3 months pre-index (*n* = 365) had similar sociodemographic, clinical and asthma-related characteristics to the overall group (Table 1).

### PROMs stratified by frequency of self-reported moderate exacerbations

**Fig. 3:**
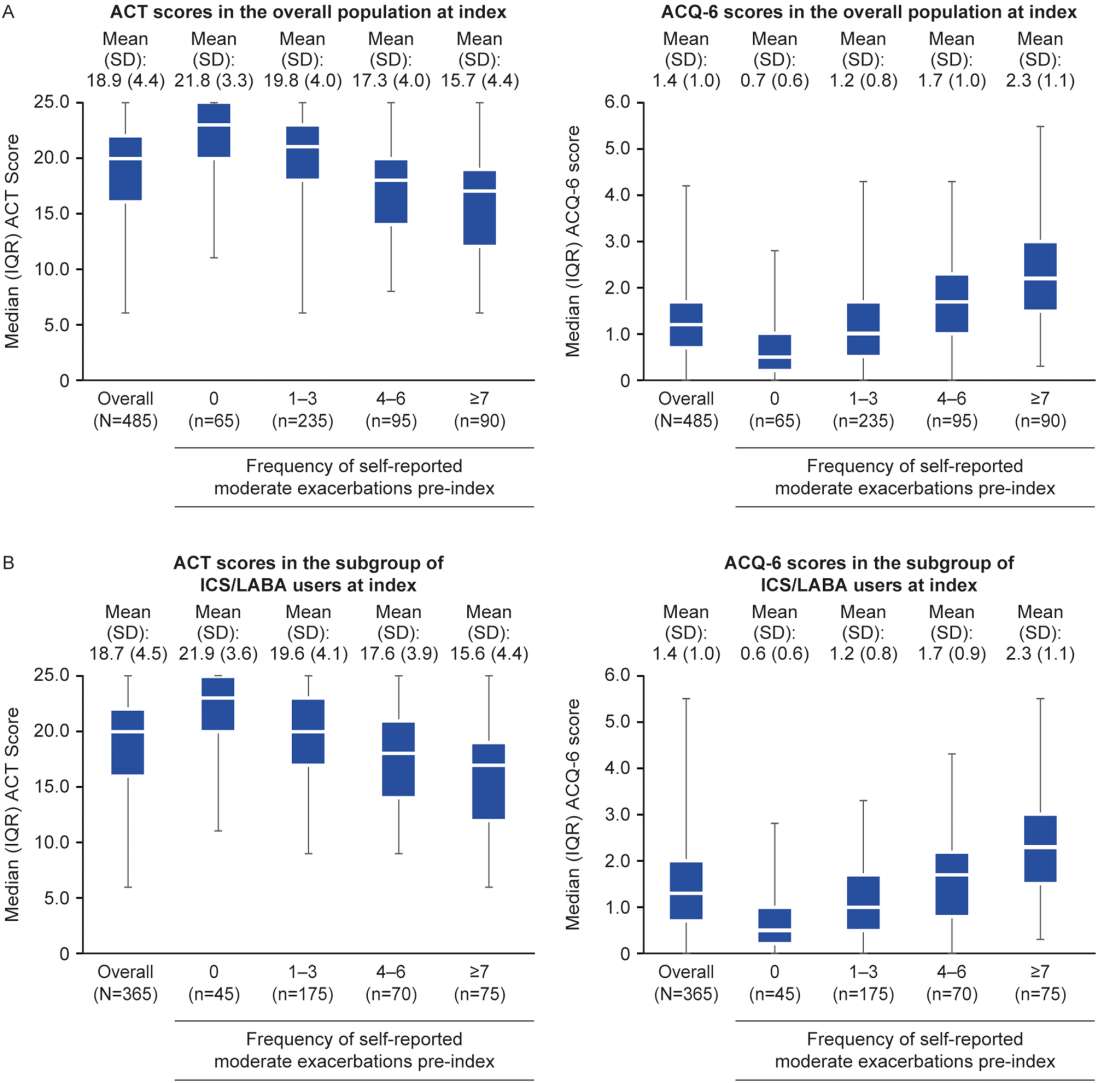
ACT and ACQ-6 scores at index in the overall population (**A**) and the subgroup of ICS/LABA users (**B**). ACQ-6, Asthma Control Questionnaire 6-item; ACT, Asthma Control Test; ICS, inhaled corticosteroid; IQR, interquartile range; LABA, long-acting β_2_-agonist; SD, standard deviation

Overall, ACT and ACQ-6 scores at the index date showed a trend towards worsening asthma control with increasing frequency of moderate exacerbations, with the lowest ACT scores and highest ACQ-6 scores reported in patients with ≥ 7 exacerbations (Fig. 3A). Similar mean (SD) asthma control scores were reported in the overall population (Fig. 3A) and the subgroup of ICS/LABA users (Fig. 3B) for ACT and ACQ (ACT: 18.9 [4.4] vs. 18.7 [4.5]; ACQ-6: 1.4 [1.0] vs. 1.4 [1.0]), and these similarities were maintained when stratified by frequency of self-reported moderate exacerbations.

Overall, the mean (SD) CASIS score was 34.3 (23.0). Patients with a greater frequency of exacerbations in the 12 months pre-index had a numerically higher mean (SD) CASIS score at index (≥ 7 exacerbations: 49.2 [23.5]), indicating a greater level of sleep impairment, compared with those with no self-reported exacerbations (19.2 [16.4]). Of patients who were ICS/LABA users in the 3 months pre-index (*n* = 365), mean (SD) CASIS score was 34.6 (23.3), similar to the overall group.

### All-cause and asthma-related HCRU and healthcare costs

#### HCRU

HCRU and healthcare costs were assessed in the secondary patient population with both 12 months pre- and post-index data (*N* = 450). For all-cause HCRU during the 12 months pre-index in the overall population, 98.9% of patients had a GP consultation (mean [SD] number of visits: 21.1 [15.4]), 61.1% required outpatient visits (mean [SD]: 2.8 [4.1]), 27.8% required inpatient visits (mean [SD]: 0.5 [1.3]) and 26.7% required A&E visits (mean [SD]: 0.5 [2.3]). All types of all-cause HCRU generally increased with increasing frequency of moderate exacerbations pre-index, although the length of stay for inpatient visits remained similar (Table 2). Similar trends were also observed for all-cause consultations in the 12 months post-index (Table 2).

**Table 2:** All-cause HCRU in the 12 months pre- and post-index

	All-cause HCRU 12 months pre-index					All-cause HCRU 12 months post-index							
	Overall	Frequency of self-reported moderate exacerbations in 12 months pre-index				Overall		Frequency of self-reported moderate exacerbations in 12 months pre-index					
	(*N* = 450)	0 (*n* = 65)	1–3 (*n* = 220)	4–6 (*n* = 85)	≥ 7 (*n* = 80)		(*N* = 450)		0 (*n* = 65)	1–3 (*n* = 220)	4–6 (*n* = 85)	≥ 7 (*n* = 80)	
**GP consultations**													
≥ 1 GP consultation, n (%)	445 (98.9)	65 (100.0)	215 (97.7)	85 (100.0)	80 (100.0)		445 (98.9)		60 (92.3)	220 (100.0)	85 (100.0)	80 (100.0)	
Mean (SD)	21.1 (15.4)	15.8 (10.1)	19.6 (13.5)	23.8 (18.4)	26.5 (17.9)		21.1 (15.3)		15.5 (10.4)	19.0 (13.8)	23.2 (14.8)	29.3 (19.1)	
**Outpatient visits**													
≥ 1 outpatient visit, n (%)	275 (61.1)	35 (53.8)	120 (54.5)	60 (70.6)	60 (75.0)		265 (58.9)		30 (46.2)	120 (54.5)	55 (64.7)	60 (75.0)	
Mean (SD)	2.8 (4.1)	2.8 (5.9)	2.4 (3.6)	3.4 (4.2)	3.2 (3.6)		2.8 (3.9)		2.1 (3.7)	2.4 (3.4)	3.2 (4.3)	4.0 (4.8)	
**Inpatient visits**													
≥ 1 inpatient visit, n (%)	125 (27.8)	15 (23.1)	55 (25.0)	25 (29.4)	30 (37.5)		125 (27.8)		< 8	60 (27.3)	25 (29.4)	35 (43.8)	
Mean (SD)	0.5 (1.3)	0.4 (0.7)	0.5 (1.4)	0.5 (1.0)	0.8 (1.7)		0.5 (1.3)		0.2 (0.5)	0.5 (1.3)	0.4 (0.7)	1.0 (1.8)	
**Cumulative length of inpatient visit (days)**													
Mean (SD)	0.6 (2.9)	0.5 (1.8)	0.6 (2.9)	0.6 (4.0)	0.5 (1.5)		0.6 (3.6)		0.0 (0.0)	0.5 (4.1)	0.5 (2.2)	1.4 (4.6)	
**A&E visits**													
≥ 1 A&E visit, n (%)	120 (26.7)	10 (15.4)	55 (25.0)	30 (35.3)	30 (37.5)		135 (30.0)		15 (23.1)	65 (29.5)	25 (29.4)	35 (43.8)	
Mean (SD)	0.5 (2.3)	0.2 (0.4)	0.4 (1.1)	1.0 (4.8)	0.6 (1.1)		0.5 (1.0)		0.3 (0.6)	0.4 (0.8)	0.5 (1.6)	0.7 (1.1)	

Results based on 1–<8 patients were suppressed, and all other counts were rounded to the nearest five to comply with HES analysis guidance [23]; consequently, proportions of patients may not total 100%

A&E, accident and emergency; GP, general practitioner; HCRU, healthcare resource utilisation; HES, Hospital Episode Statistics; SD, standard deviation

For asthma-related HCRU during the 12 months pre-index in the overall population, 95.6% of patients had an asthma-related GP consultation (mean [SD]: 4.2 [3.5]), with 10.0% of patients requiring asthma-related outpatient visits and 22.2% requiring asthma-related inpatient visits; data on A&E visits were suppressed due to a low sample size (Table 3). As with all-cause HCRU, asthma-related HCRU generally increased with moderate exacerbation frequency pre-index; more patients in the ≥ 7 moderate exacerbation group had asthma-related inpatient visits in the 12 months pre-index (31.3%, *n* = 25/80), compared with the 4–6 (23.5%, *n* = 20/85), 1–3 (18.2%, *n* = 40/220) and no exacerbation (15.4%, *n* = 10/65) groups (Table 3). Similar trends were observed for asthma-related GP consultations, and outpatient and inpatient visits in the 12 months post-index (Table 3).

**Table 3:** Asthma-related HCRU in the 12 months pre- and post-index

	Asthma-related HCRU 12 months pre-index					Asthma-related HCRU 12 months post-index				
	Overall	Frequency of self-reported moderate exacerbations in 12 months pre-index				Overall	Frequency of self-reported moderate exacerbations in 12 months pre-index			
	(*N* = 450)	0 (*n* = 65)	1–3 (*n* = 220)	4–6 (*n* = 85)	≥ 7 (*n* = 80)	(*N* = 450)	0 (*n* = 65)	1–3 (*n* = 220)	4–6 (*n* = 85)	≥ 7 (*n* = 80)
**GP consultations**										
≥ 1 GP consultation, n (%)	430 (95.6)	60 (92.3)	210 (95.5)	80 (94.1)	80 (100.0)	425 (94.4)	60 (92.3)	210 (95.5)	80 (94.1)	80 (100.0)
Mean (SD)	4.2 (3.5)	3.2 (2.8)	4.0 (2.9)	4.4 (3.7)	5.4 (4.6)	3.9 (3.0)	2.8 (2.0)	3.5 (2.5)	4.1 (2.8)	5.5 (4.0)
**Outpatient visits**										
≥ 1 outpatient visit, n (%)	45 (10.0)	< 8	20 (9.1)	15 (17.6)	10 (12.5)	50 (11.1)	< 8	20 (9.1)	10 (11.8)	15 (18.8)
Mean (SD)	0.2 (0.9)	0.2 (1.0)	0.2 (0.7)	0.2 (0.6)	0.4 (1.5)	0.2 (0.8)	0.1 (0.4)	0.1 (0.4)	0.2 (0.8)	0.5 (1.4)
**Inpatient visits**										
≥ 1 inpatient visit, n (%)	100 (22.2)	10 (15.4)	40 (18.2)	20 (23.5)	25 (31.3)	95 (21.1)	< 8	45 (20.5)	15 (17.6)	25 (31.3)
Mean (SD)	0.4 (1.1)	0.2 (0.5)	0.3 (1.1)	0.4 (1.0)	0.7 (1.6)	0.4 (1.1)	0.1 (0.5)	0.4 (1.1)	0.2 (0.6)	0.7 (1.6)
**Cumulative length of inpatient visit (days)**										
Mean (SD)	0.4 (2.2)	0.3 (1.0)	0.3 (1.5)	0.6 (4.0)	0.4 (1.4)	0.4 (2.1)	0.0 (0.0)	0.2 (1.2)	0.3 (1.8)	1.1 (4.0)
**A&E visits**										
≥ 1 A&E visit, n (%)	< 8	0 (0.0)	< 8	< 8	< 8	< 8	0 (0.0)	< 8	0 (0.0)	< 8
Mean (SD)	0.016 (0.124)	0 (0.0)	0.005 (0.0681)	0.047 (0.212)	0.025 (0.156)	0.004 (0.0671)	0.0 (0.0)	0.005 (0.0681)	0.0 (0.0)	0.012 (0.111)

Results based on 1–<8 patients were suppressed, and all other counts were rounded to the nearest five to comply with HES analysis guidance [23]; consequently, proportions of patients may not total 100%

A&E, accident and emergency; GP, general practitioner; HCRU, healthcare resource utilisation; HES, Hospital Episode Statistics; SD, standard deviation

#### Healthcare costs

**Fig. 4:**
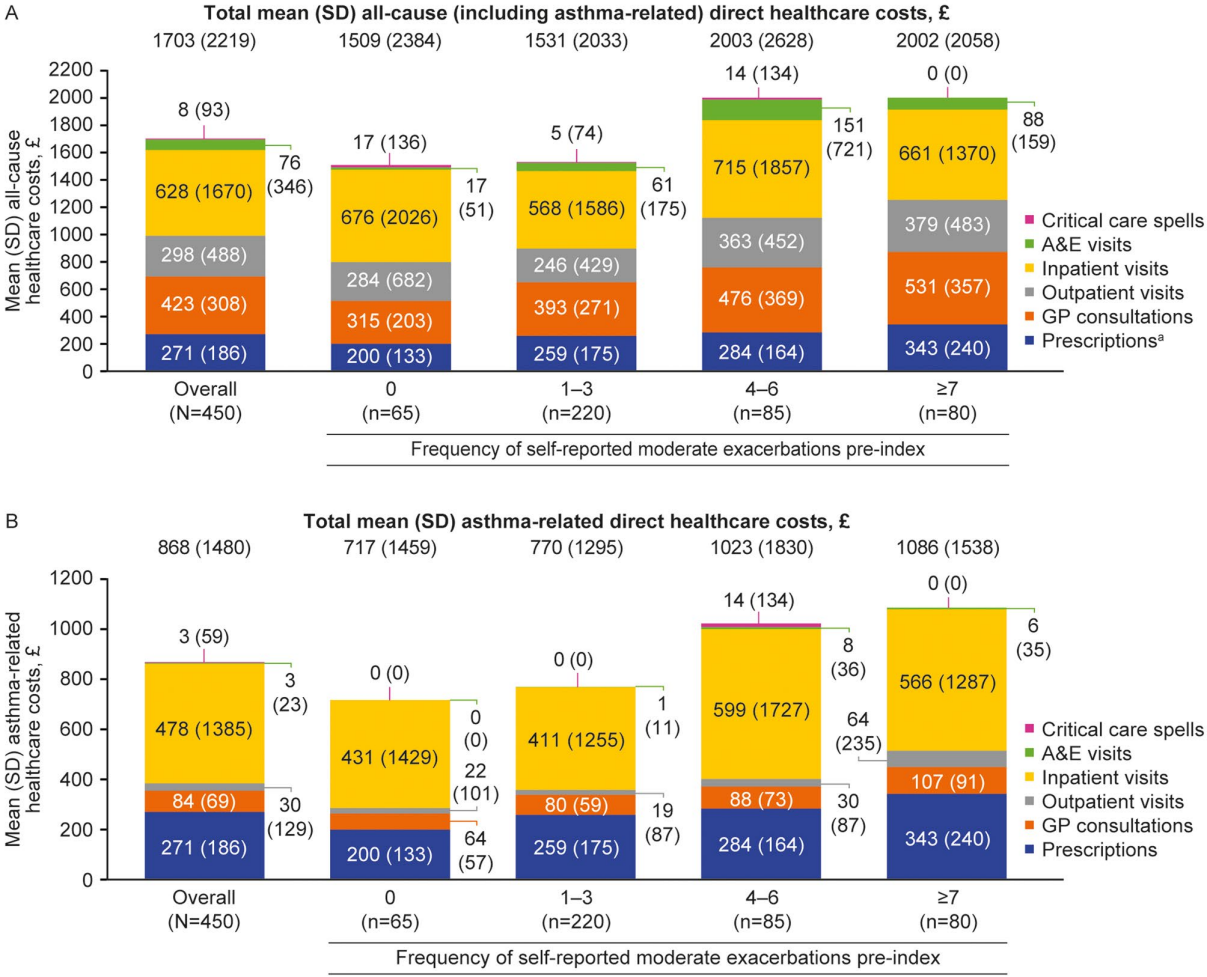
All-cause (**A**) and asthma-related (**B**) healthcare costs pre-index, stratified by frequency of self-reported moderate exacerbations. Self-reported moderate exacerbations were assessed during the 12-month period pre-index (date of consent to inclusion in the Ext-SLS). ^a^Asthma-related prescription costs are reported as all-cause prescription costs as other prescription costs data were not collected. A&E, accident and emergency; Ext-SLS, Extended Salford Lung Study; GP, general practitioner; SD, standard deviation

During the 12 months pre-index, mean (SD) total all-cause and asthma-related costs in the overall population (*N* = 450) were £1703 (£2219) and £868 (£1480), respectively (Fig. 4). When stratified by frequency of self-reported moderate exacerbations pre-index, mean (SD) total all-cause and asthma-related costs were lowest in patients with no moderate exacerbations (£1509 [£2384] and £717 [£1459], respectively), and increased with more frequent moderate exacerbations, rising to £2002 (£2058) and £1086 (£1538), respectively, for patients with ≥ 7 moderate exacerbations (Fig. 4).

**Fig. 5:**
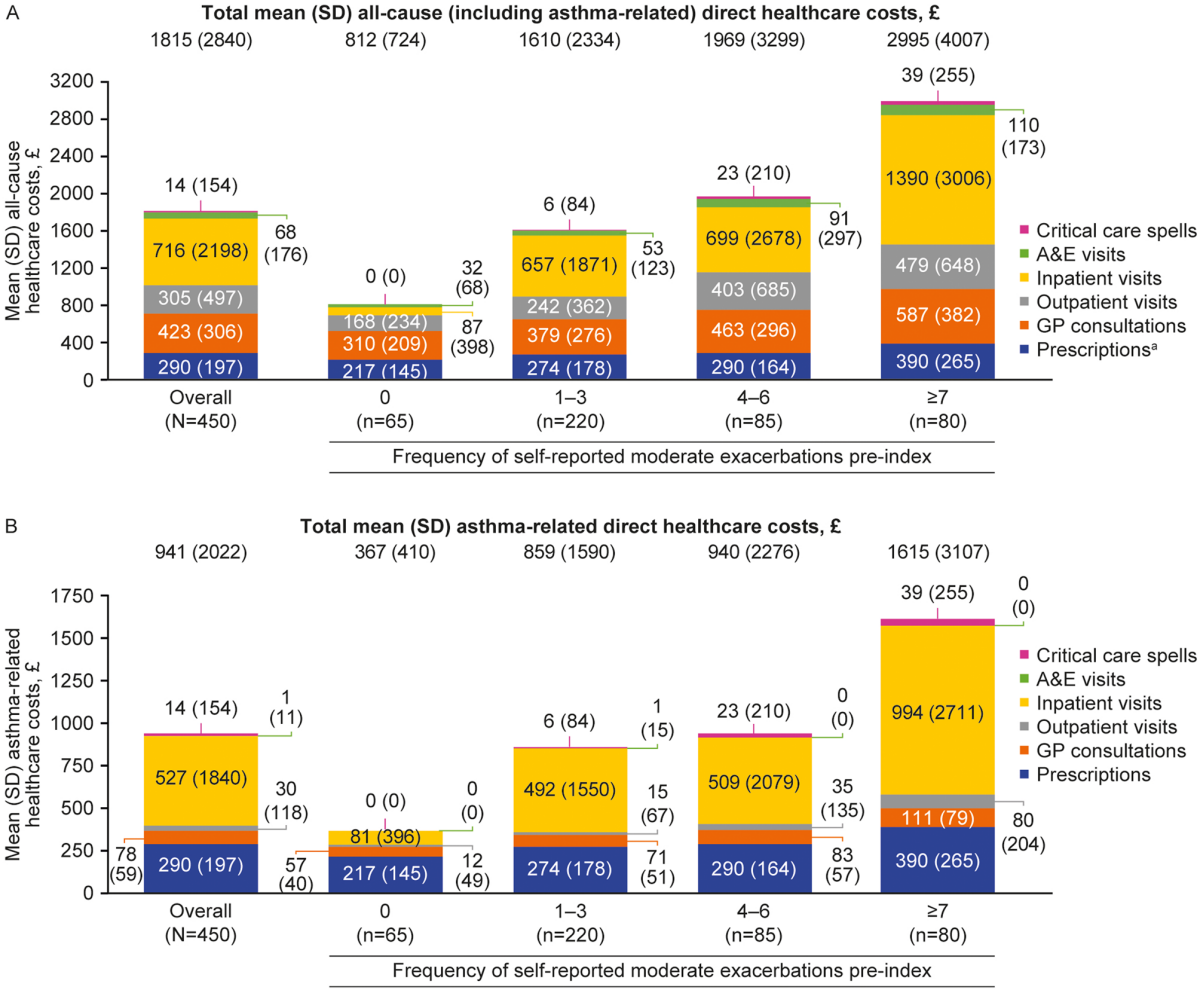
All-cause (**A**) and asthma-related (**B**) healthcare costs post-index, stratified by frequency of self-reported moderate exacerbations. Self-reported moderate exacerbations were assessed during the 12-month period pre-index (date of consent to inclusion in the Ext-SLS). ^a^Asthma-related prescription costs are reported as all-cause prescription costs as other prescription costs data were not collected. A&E, accident and emergency; Ext-SLS, Extended Salford Lung Study; GP, general practitioner; SD, standard deviation

During the 12 months post-index, mean (SD) total all-cause and asthma-related costs for the overall population were £1815 (£2840) and £941 (£2022), respectively (Fig. 5). Total all-cause and asthma-related costs rose incrementally with higher exacerbation frequency. Among patients with ≥ 7 moderate exacerbations, mean (SD) total all-cause and asthma-related costs were £2995 (£4007) and £1615 (£3107), respectively, compared with £812 (£724) and £367 (£410), respectively, for patients with no moderate exacerbations (Fig. 5).

## Discussion

In this real-world retrospective observational study, approximately only one out of ten symptomatic patients with asthma reported no moderate exacerbations over a 1-year period, with approximately 40% of patients experiencing four events or more. These trends were generally consistent across patient medication subgroups, even in patients who had been prescribed ICS/LABA therapy. As the frequency of pre-index moderate exacerbations increased, more patients had poor asthma control and were more likely to experience comorbidities or come from a more deprived area. Patients were also more likely to have higher reliever medication use, an expected finding based on the definition of moderate exacerbations used here which includes an increase in reliever use, and is consistent with previous studies [24, 25]. Monitoring of SABA is therefore a potential mechanism to identify patients with high disease burden requiring medication and disease management review who may benefit from stepping-up therapy. Additionally, greater HCRU and direct healthcare costs were incurred with increasing moderate exacerbation history. Overall, these results indicate a significant burden of moderate exacerbations and a need for holistic management review and better treatment optimisation.

Of the patients included in this study, 87% self-reported having ≥ 1 moderate exacerbation in the previous year. This is substantially higher than previously reported in a UK-based retrospective database study using an algorithmic approach, which found that only 36% of patients experienced any type of exacerbation (including both moderate or severe) over a 7-year period [26]. These differences in the proportion of patients experiencing moderate exacerbations may in part reflect the difficulty in defining and monitoring these events. Although moderate exacerbations are defined explicitly by the UK National Institute for Clinical Excellence as events with a PEFR > 50–75% of predicted [27], PEFR is rarely monitored in clinical practice, limiting the practicality of this definition to define exacerbations in real-world situations. Additionally, the Global Initiative for Asthma defines moderate exacerbations alongside mild events [1]. Together, this highlights a need to implement a consistent definition of moderate exacerbations, as previously outlined in a joint American Thoracic Society-European Respiratory Society statement [28], which considers routinely used and accessible clinical measures to ensure consistent monitoring between healthcare practices.

Patients in the current study with a history of more frequent moderate exacerbations had poorer asthma control at index compared with those with fewer exacerbations. It should be noted that as asthma control was assessed using the ACQ-6 and ACT, parts of which assess patients’ rescue medication use, and moderate exacerbations were defined in this study based on symptom worsening requiring rescue medication, this may confound the results. Investigating the relationship between asthma control and exacerbations is a possible focus of future work. Indeed, previous studies have identified that poorer asthma control can increase the risk of future exacerbation events [7, 8, 29]. A previous prospective study by Gutiérrez et al. found that moderate exacerbations, in addition to ACT score, are significant predictors of future moderate exacerbations [8]. This is similar to the increased risk of future severe exacerbations following a previous exacerbation as demonstrated by Chipps et al., which were increased almost four-fold even when adjusted for asthma control [29]. Additionally, Lane et al. demonstrated that in over one-third of patients with a severe exacerbation, a moderate exacerbation preceded the severe event, suggesting that managing moderate exacerbations may reduce the risk of experiencing more severe exacerbations, leading to better outcomes [7]. This highlights the importance of monitoring moderate exacerbations more closely to identify patients who may be at risk of severe exacerbations and may therefore benefit from treatment step up to prevent future events. This also emphasises the importance of maintaining good asthma control to prevent a vicious circle of increased risk of further exacerbations, to help prevent poor disease outcomes, which can be aided by increased use of measures such as the ACT and ACQ-6 in the clinic.

Asthma control and reducing exacerbations are important, long-standing goals in the management of asthma [1], and there has been a recent call for a ‘zero tolerance’ approach to exacerbations, with the aim to reduce exacerbations by 50% from 2018 levels [30]. In the present study, due to the descriptive nature of the analyses, we were unable to fully explore the causality between asthma control status and moderate exacerbations. Additionally, the proportion of patients experiencing asthma-related inpatient hospital visits increased as the number of reported moderate exacerbations increased. This suggests a link between moderate exacerbations and increasing likelihood of severe exacerbations [7], further confounding the exploration of causality between moderate exacerbations and asthma control with this data as the impact of moderate versus severe exacerbations was not assessed. However, it is likely that improving asthma control or reducing exacerbations is likely to result in improvements in the other [31]. Therefore, as moderate exacerbations may be under-recognised [28, 32], deteriorations in asthma control or recurrence of symptoms may provide a useful trigger to identify patients who may be experiencing frequent moderate exacerbations and require a step-up in their medication to improve overall disease control.

When stratified by asthma treatment class at index in the current study, moderate exacerbations in the previous 12 months were reported by 66.7–100.0% of patients, with a greater proportion of patients reporting exacerbations receiving ICS plus add-on therapy (LABA or LTRA + LABA/LAMA) compared with ICS-only maintenance therapy. Although the sample sizes for some treatment groups were low, and results should therefore be interpreted with caution, this suggests a continued high burden of disease despite the use of multiple maintenance therapies including ICS/LABA. Overall, these results indicate that a large proportion of patients experiencing moderate exacerbations may benefit from their medication being reviewed, in addition to assessment for other factors that may contribute to poor symptom control. Adherence to medication may be improved with patient education, as well as with the use of shared decision making/motivational communication between patients and physicians, leading to improved clinical outcomes [33–35]. Improvements in inhaler technique and reducing exposure to environmental triggers can also improve asthma control [1]. Factors such as the presence of comorbidities and living in a more deprived area also negatively affect asthma control in patients, highlighting the extent to which disease management must reach [36, 37]. Holistic reviews of management may therefore also benefit patients alongside medication reviews.

The burden of frequent moderate exacerbations is further highlighted by HCRU and cost data. Previous evidence has suggested that all forms of asthma-related HCRU are significantly increased in patients with one or more versus no moderate/severe exacerbations, and that direct all-cause healthcare costs increase by a third [38]. The current study’s data build on these results, focusing solely on moderate events and showing that all-cause and asthma-related HCRU and direct healthcare costs increase substantially from patients with no moderate exacerbations in the previous year to those with seven or more; these trends were most pronounced during the 12 months post-index, with all-cause total costs increasing four-fold from approximately £800 to £3000. Overall, these results suggest that reducing the frequency of moderate exacerbations may allow for reductions in HCRU and costs.

There are several strengths of this study, including the use of multiple connected data sources (primary and secondary care and additional demographic and clinical data not routinely available), allowing for the observation of the full patient journey. Other strengths include this being one of the first studies to use a secondary data source to describe the frequency of moderate exacerbations in a real-world population of patients with asthma, strengthened by the use of self-reporting methodology, which allowed the observation of moderate exacerbation events that EMR-based algorithms may be insensitive to. Despite these strengths, there are limitations that should be considered when interpreting these results. Self-reporting of moderate exacerbations in the previous year was subject to recall bias, and patients may have inaccurately recalled the number of relevant exacerbations they had experienced. The definition of moderate exacerbations used (an event requiring increased use of rescue medication, but that also does not require a hospital visit) may have been subject to different interpretations, leading to some variation in how patients responded. The number of days of SABA use was not available in the prescription information from primary care records and so the impact of the reported moderate exacerbations could not be assessed. Additionally, the sample size for some of the subgroups included in this study were small, so results should be interpreted with caution. Finally, due to the source data, medication use reflected prescription data rather than actual medication use, so it was impossible to know whether the medication was dispensed and being administered as it was prescribed. For the medication-use estimates, prescriptions were assumed to cover 30 days of use, whereas for some medications the coverage may have been shorter.

## Conclusion

This study has shown that moderate exacerbations are prevalent in a real-world population of patients with asthma, and that an increased frequency of these events is associated with poor symptom control and increased HCRU and costs. The high prevalence of moderate exacerbations was observed despite patients receiving ICS/LABA-based therapy, suggesting that the clinical significance and burden of these events may be under-recognised, and indicates a potential unmet need for increased monitoring of moderate exacerbations to identify patients who may benefit from step up asthma medication, as well as for a holistic review of disease management.

## Electronic supplementary material

Below is the link to the electronic supplementary material.

Supplementary Material 1: Figure S1 Study design

Supplementary Material 2: Figure S2 Patient attrition

Supplementary Material 3: Figure S3 Sensitivity analyses of self-reported moderate asthma exacerbations (pre-index), by maintenance treatment class at index

Supplementary Material 4: Figure S4 Sensitivity analyses of self-reported moderate asthma exacerbations (pre-index), by asthma control status (ACT and ACQ-6) at index

## Data Availability

The data are provided by patients and collected by the UK National Health Service as part of their care and support. The interpretation and conclusions contained in this study are those of the author/s alone. Data from HES Copyright© (2023), re-used with the permission of the Health and Social Care Information Centre. All rights reserved. Authors had access to the study data for the purposes of this work only. Data were accessed through an existing GSK license to address prespecified research questions only. Therefore, the data cannot be broadly disclosed or made publicly available at this time. Access to each database can be requested via the respective websites (EMIS Web and Vision electronic patient record systems and NHS Digital).Competing interests.

## Abbreviations

ACQ-6: Asthma Control Questionnaire 6-item
ACT: Asthma Control Test
A&E: Accident and Emergency
BMI: Body Mass Index
CASIS: Chronic Obstructive Pulmonary Disease and Asthma Sleep Impact Scale
COPD: Chronic Obstructive Pulmonary Disease
EMR: Electronic Medical Record
Ext-SLS: Extended Salford Lung Study
GP: General Practitioner
HCRU: Healthcare Resource Utilisation
HES: Hospital Episode Statistics
HRG: Healthcare Resource Group
ICS: Inhaled Corticosteroid
IQR: Interquartile Range
LABA: Long-Acting β_2_-Agonist
LAMA: Long-Acting Muscarinic Antagonist
LTRA: Leukotriene Receptor Antagonist
MITT: Multiple-Inhaler Triple Therapy
NHS: National Health Service
OCS: Oral Corticosteroid
PDE4: Phosphodiesterare-4
PEFR: Peak Expiratory Flow Rate
PROM: Patient-Reported Outcome Measure
SABA: Short-Acting β_2_-Agonist
SABD: Short-Acting Bronchodilator
SD: Standard Deviation
SITT: Single-Inhaler Triple Therapy
SLS: Salford Lung Study
UK: United Kingdom
