# Supplementary material for: Describing the burden of moderate exacerbations in patients with asthma from the Extended Salford Lung Study (Ext-SLS): a retrospective cohort study
Source: Respir Res. 2025 Mar 29;26:121. doi: 10.1186/s12931-025-03199-5 (PMC11955143; doi:10.1186/s12931-025-03199-5)
Supplement: Supplementary file 1 — Supplementary Material 1: Figure S1 Study design [file 12931_2025_3199_MOESM1_ESM.docx]

**Additional file 1**

**
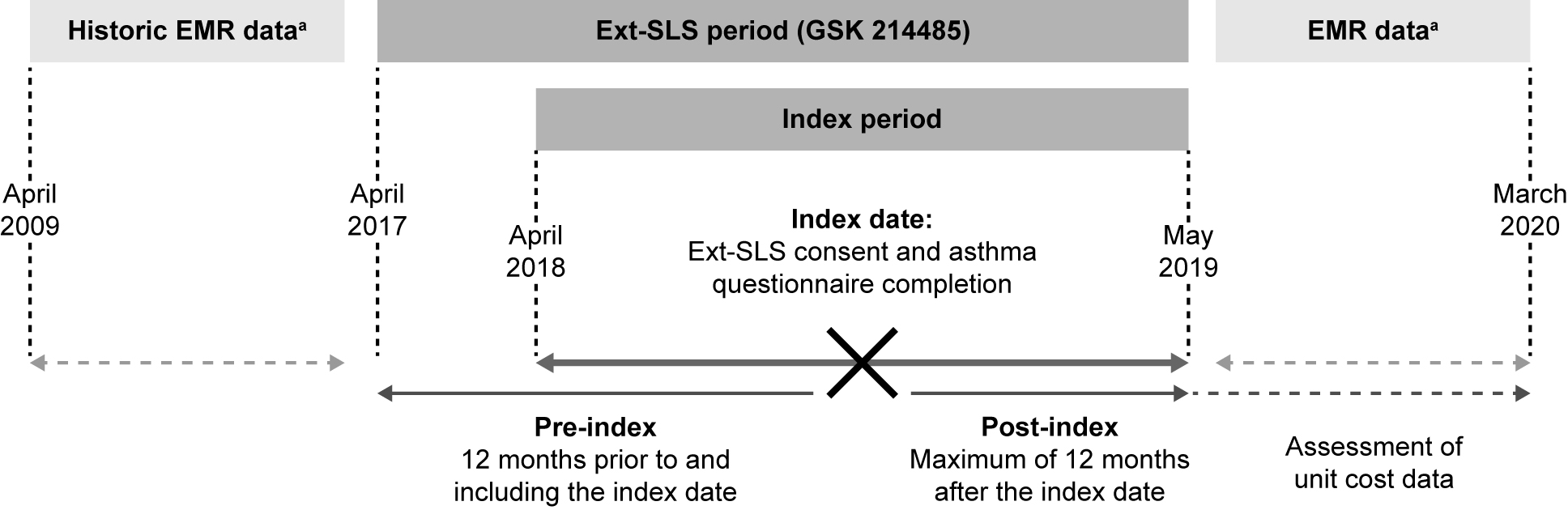
Figure S1.** Study design

^a^Primary and secondary care data (for approximately 10 years in total, up to and including December 2019 and March 2020, respectively) were collected for consenting patients, and an asthma-related questionnaire was completed on the index date to capture detailed information not routinely or robustly available in EMR data, such as PROMs.

EMR, electronic medical record; Ext-SLS, Extended Salford Lung Study; PROM, patient-reported outcome measure.
