# Supplementary material for: Describing the burden of moderate exacerbations in patients with asthma from the Extended Salford Lung Study (Ext-SLS): a retrospective cohort study
Source: Respir Res. 2025 Mar 29;26:121. doi: 10.1186/s12931-025-03199-5 (PMC11955143; doi:10.1186/s12931-025-03199-5)
Supplement: Supplementary file 2 — Supplementary Material 2: Figure S2 Patient attrition [file 12931_2025_3199_MOESM2_ESM.docx]

**Additional file 2**

**
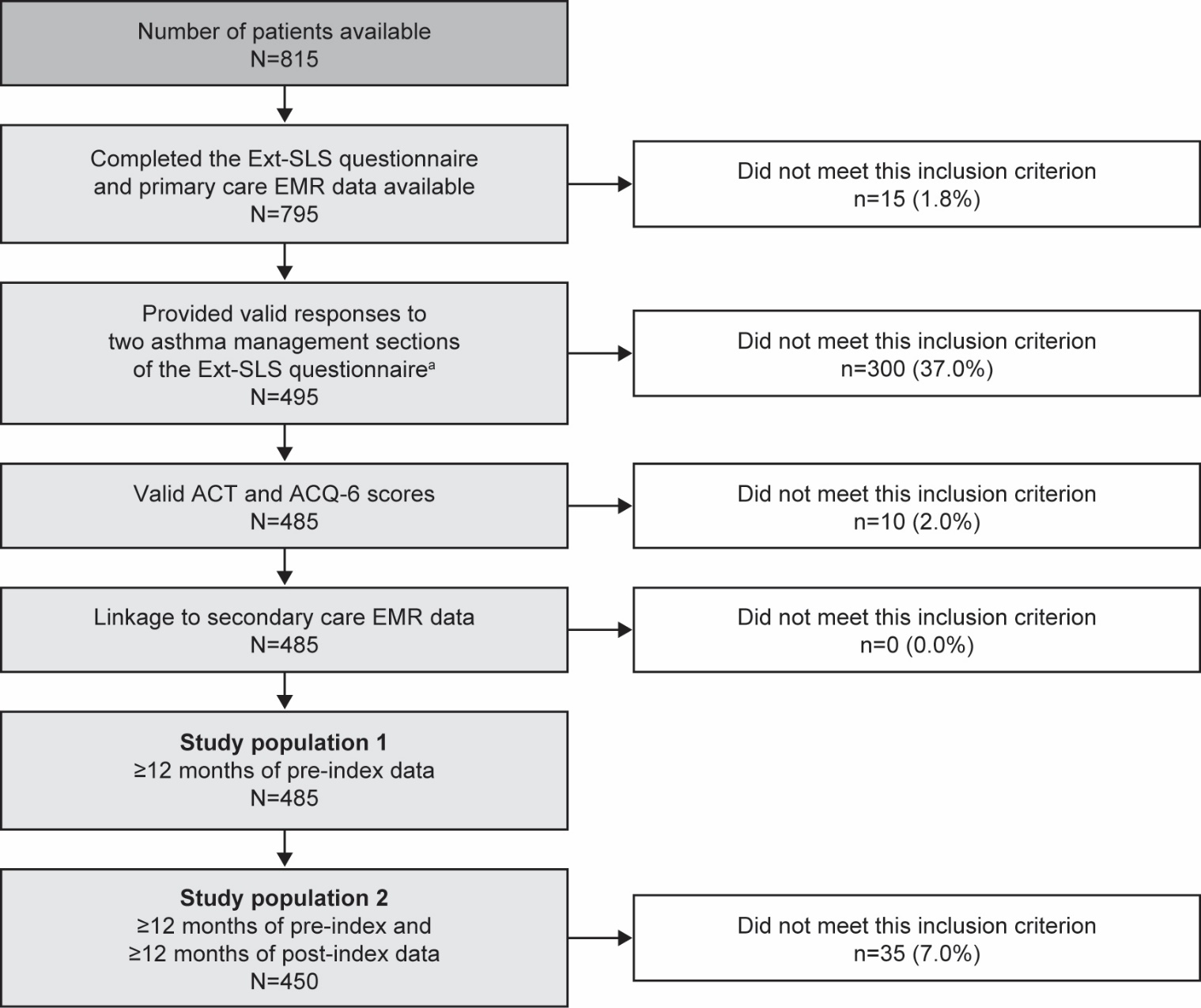
Figure S2.** Patient attrition

Patient numbers were rounded to the nearest 5 and values <8 were suppressed to comply with HES analysis guidance [1]; ^a^Number of extra inhalations taken during a moderate asthma exacerbation and number of moderate asthma exacerbations in the previous year.

ACQ-6, Asthma Control Questionnaire 6-item; ACT, Asthma Control Test; EMR, electronic medical record; Ext-SLS, Extended Salford Lung Study; HES, Hospital Episode Statistics.

**References**

1. NHS Digital. Hospital Episode Statistics (HES). https://digital.nhs.uk/data-and-information/data-tools-and-services/data-services/hospital-episode-statistics. Accessed: 07 July 2023.
