# Supplementary material for: Describing the burden of moderate exacerbations in patients with asthma from the Extended Salford Lung Study (Ext-SLS): a retrospective cohort study
Source: Respir Res. 2025 Mar 29;26:121. doi: 10.1186/s12931-025-03199-5 (PMC11955143; doi:10.1186/s12931-025-03199-5)
Supplement: Supplementary file 3 — Supplementary Material 3: Figure S3 Sensitivity analyses of self-reported moderate asthma exacerbations (pre-index), by maintenance treatment class at index [file 12931_2025_3199_MOESM3_ESM.docx]

**Additional file 3**


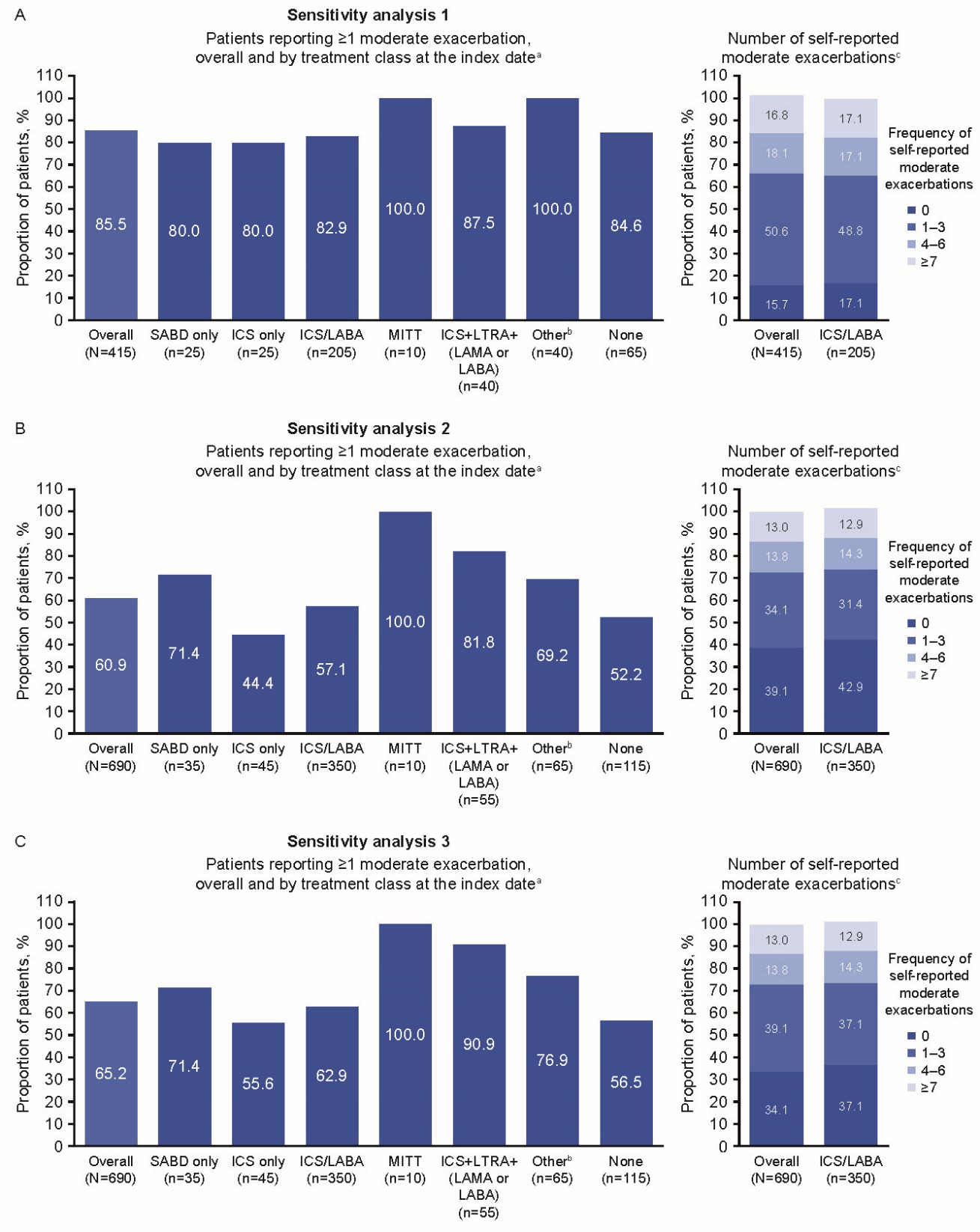
**Figure S3.** Sensitivity analyses of self-reported moderate asthma exacerbations (pre-index), by maintenance treatment class at index

Three sensitivity analyses were performed on the primary outcome (number of self-reported moderate asthma exacerbations in the 12 months pre-index). The first excluded patients with comorbid COPD (determined by the presence of diagnosis codes pre-index [inclusive]). The second and third sensitivity analyses addressed data from those with an invalid response in the Ext-SLS questionnaire for the number of extra inhalations taken during a moderate asthma exacerbation. For how often this occurred during the previous 12 months, the second analysis imputed a value of 0 whereas the third analysis imputed values of 0 or 1–3 for patients with ACT scores that indicated “controlled” symptoms (ACT >19) and “not controlled” symptoms (ACT ≤19), respectively. Results based on 1–<8 patients were suppressed, and all other counts were rounded to the nearest five to comply with HES analysis guidance [1]; consequently, proportions of patients may not total 100%.

^a^Data were unavailable for ICS+LTRA, SITT, and ICS/LABA+LAMA+LTRA due to data suppression; ^b^Including OCS monotherapy, methylxanthines and PDE4 inhibitors, as well as non-standard asthma maintenance regimens including ICS, LABA or LTRA monotherapy, and combinations of ICS, LABA, LAMA, LTRA, OCS, SABD and xanthines. Free/open combination therapy was identified when there was ≥1 day of overlap between all components; ^c^ICS/LABA use was defined as a prescription of fixed-dose combination ICS/LABA at index (±15 days).

ACT, Asthma Control Test; COPD, chronic obstructive pulmonary disease; Ext-SLS, Extended Salford Lung Study; HES, Hospital Episode Statistics; ICS, inhaled corticosteroid; LABA, long-acting β_2_-agonist; LAMA, long-acting muscarinic antagonist; LTRA, leukotriene receptor antagonist; MITT, multi-inhaler triple therapy; OCS, oral corticosteroid; PDE4, phosphodiesterase-4; SABD, short-acting bronchodilator; SITT, single-inhaler triple therapy.
