# Supplementary material for: Describing the burden of moderate exacerbations in patients with asthma from the Extended Salford Lung Study (Ext-SLS): a retrospective cohort study
Source: Respir Res. 2025 Mar 29;26:121. doi: 10.1186/s12931-025-03199-5 (PMC11955143; doi:10.1186/s12931-025-03199-5)
Supplement: Supplementary file 4 — Supplementary Material 4: Figure S4 Sensitivity analyses of self-reported moderate asthma exacerbations (pre-index), by asthma control status (ACT and ACQ-6) at index [file 12931_2025_3199_MOESM4_ESM.docx]

**Additional file 4**


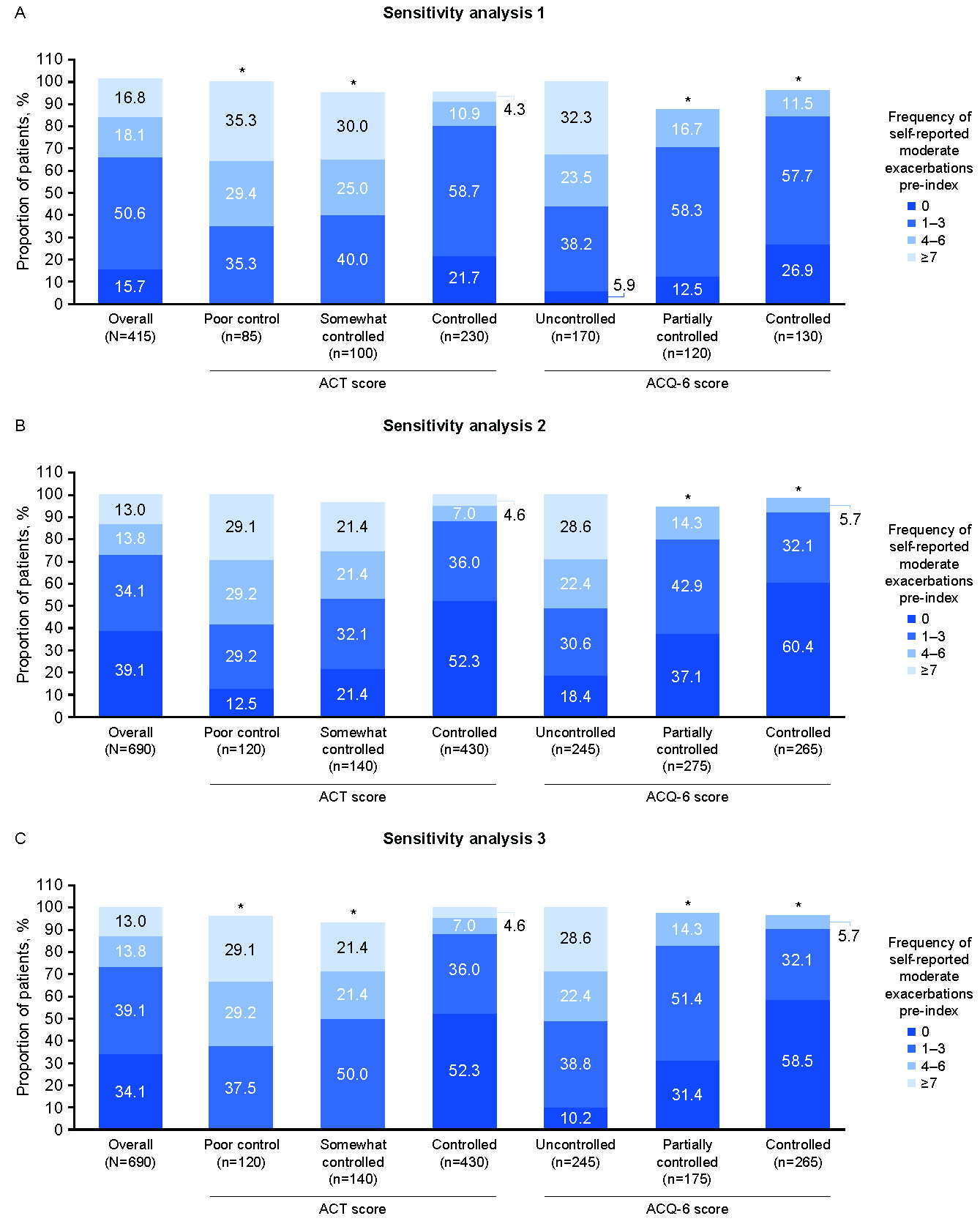
**Figure S4.** Sensitivity analyses of self-reported moderate asthma exacerbations (pre-index), by asthma control status (ACT and ACQ-6) at index

Three sensitivity analyses were performed on the primary outcome (number of self-reported moderate asthma exacerbations in the 12 months pre-index). Sensitivity analysis 1: excluding patients with comorbid COPD and those with an invalid response in the Ext-SLS questionnaire for the number of extra inhalations taken during a moderate asthma exacerbation; sensitivity analysis 2: where patients had values of 0 imputed for how often this occurred during the previous 12 months; sensitivity analysis 3: where patients had values of 0 imputed for ACT scores that indicated “controlled” symptoms (ACT >19), and 1–3 when ACT scores indicated “not controlled” symptoms (ACT ≤19). Results based on 1–<8 patients were suppressed (denoted by an asterisk), and all other counts were rounded to the nearest five to comply with HES analysis guidance [1]; consequently, proportions of patients may not total 100%. ACT score: poor control (<16), somewhat controlled (16–19), and controlled (>19). ACQ-6 score: uncontrolled (≥1.50), partially controlled (>0.75 to <1.5), and controlled (≤0.75).

ACQ-6, Asthma Control Questionnaire 6-item; ACT, Asthma Control Test; COPD, chronic obstructive pulmonary disease; Ext-SLS, Extended Salford Lung Study; HES, Hospital Episode Statistics.

**References**

1. NHS Digital. Hospital Episode Statistics (HES). https://digital.nhs.uk/data-and-information/data-tools-and-services/data-services/hospital-episode-statistics. Accessed: 07 July 2023.
